# Incidence of Cerebral Venous Thrombosis Following SARS-CoV-2 Infection vs mRNA SARS-CoV-2 Vaccination in Singapore

**DOI:** 10.1001/jamanetworkopen.2022.2940

**Published:** 2022-03-17

**Authors:** Tian Ming Tu, Shen Jia Yi, Jasmine Shimin Koh, Seyed Ehsan Saffari, Rebecca Hui Min Hoe, Geraldine Jiangyan Chen, Hui Jin Chiew, Carol Huilian Tham, Christopher Ying Hao Seet, Ming Hui Yong, Kok Pin Yong, Andrew Che-Fai Hui, Bingwen Eugene Fan, Benjamin Yong-Qiang Tan, Amy May Lin Quek, Raymond Chee Seong Seet, Leonard Leong Litt Yeo, Kevin Tan, Umapathi N. Thirugnanam

**Affiliations:** 1 Department of Neurology, National Neuroscience Institute, Singapore; 2 Centre of Quantitative Medicine, Duke-NUS Medical School, Singapore; 3 Department of Neurology, National Neuroscience Institute, Singapore; 4 Clinical Trials and Research Unit, National Neuroscience Institute, Singapore.; 5 Department of Hematology, Tan Tock Seng Hospital; 6 Division of Neurology, Department of Medicine, National University Health System, Singapore

## Abstract

**Importance:**

Reports of cerebral venous thrombosis (CVT) after messenger RNA (mRNA)-based SARS-CoV-2 vaccination has caused safety concerns, but CVT is also known to occur after SARS-CoV-2 infection. Comparing the relative incidence of CVT after infection vs vaccination may provide a better perspective of this complication.

**Objective:**

To compare the incidence rates and clinical characteristics of CVT following either SARS-CoV-2 infection or mRNA-based SARS-CoV-2 vaccines.

**Design, Setting, and Participants:**

Between January 23, 2020, and August 3, 2021, this observational cohort study was conducted at all public acute hospitals in Singapore, where patients hospitalized with CVT within 6 weeks of SARS-CoV-2 infection or after mRNA-based SARS-CoV-2 vaccination (BNT162b2 [Pfizer-BioNTech] or mRNA-1273 [Moderna]) were identified. Diagnosis of SARS-CoV-2 infection was based on quantitative reverse transcription-polymerase chain reaction or positive serology. National SARS-CoV-2 infection data were obtained from the National Centre for Infectious Disease, Singapore, and vaccination data were obtained from the National Immunisation Registry, Singapore.

**Exposures:**

SARS-CoV-2 infection or mRNA-based SARS-CoV-2 vaccines.

**Main Outcomes and Measures:**

Clinical characteristics, crude incidence rate (IR), and incidence rate ratio (IRR) of CVT after SARS-CoV-2 infection and after mRNA SARS-CoV-2 vaccination.

**Results:**

Among 62 447 individuals diagnosed with SARS-CoV-2 infections included in this study, 58 989 (94.5%) were male; the median (range) age was 34 (0-102) years; 6 CVT cases were identified (all were male; median [range] age was 33.5 [27-40] years). Among 3 006 662 individuals who received at least 1 dose of mRNA-based SARS-CoV-2 vaccine, 1 626 623 (54.1%) were male; the median (range) age was 50 (12-121) years; 9 CVT cases were identified (7 male individuals [77.8%]; median [range] age: 60 [46-76] years). The crude IR of CVT after SARS-CoV-2 infections was 83.3 per 100 000 person-years (95% CI, 30.6-181.2 per 100 000 person-years) and 2.59 per 100 000 person-years (95% CI, 1.19-4.92 per 100 000 person-years) after mRNA-based SARS-CoV-2 vaccination. Six (66.7%) received BNT162b2 (Pfizer-BioNTech) vaccine and 3 (33.3%) received mRNA-1273 (Moderna) vaccine. The crude IRR of CVT hospitalizations with SARS-CoV-2 infection compared with those who received mRNA SARS-CoV-2 vaccination was 32.1 (95% CI, 9.40-101; *P* < .001).

**Conclusions and Relevance:**

The incidence rate of CVT after SARS-CoV-2 infection was significantly higher compared with after mRNA-based SARS-CoV-2 vaccination. CVT remained rare after mRNA-based SARS-CoV-2 vaccines, reinforcing its safety.

## Key Points

**Question:**

What is the risk of cerebral venous thrombosis (CVT) after diagnosis of SARS-CoV-2 infection compared with after messenger RNA (mRNA)-based SARS-CoV-2 vaccination?

**Findings:**

In this observational cohort study of 62 447 individuals with SARS-CoV-2 infection and 3 006 662 individuals who received mRNA-based SARS-CoV-2 vaccine in Singapore from January 23, 2020, to August 3, 2021, the incidence rate ratio of CVT requiring hospitalization within 6 weeks of SARS-CoV-2 infection was 32 times higher compared with after mRNA-based SARS-CoV-2 vaccination.

**Meaning:**

These findings suggest that the risk of CVT after SARS-CoV-2 infection is higher than after mRNA-based SARS-CoV-2 vaccination.

## Teaser

This cohort study examines the incidence rates and clinical characteristics of cerebral venous thrombosis (CVT) following either SARS-CoV-2 infection or mRNA-based SARS-CoV-2 vaccination.

## Introduction

Cerebral venous thrombosis (CVT) is a rare but important complication after COVID-19.^1,2^ In a prospective study involving 47 572 patients with COVID-19, 4 young patients (aged 27 to 38 years) were diagnosed with CVT,^3^ and CVT occurred more often in COVID-19 infection than the general population.^4^ More recently, as vaccination against SARS-CoV-2 is used as a public health measure against the COVID-19 pandemic,^5^ CVT has been reported following vaccination, mostly with the adenovirus vector-based vaccines (Ad26.COV2.S [Janssen/Johnson & Johnson]^6^ and ChAdOx1-S [Oxford-AstraZeneca]^7^). CVT after messenger RNA (mRNA)-based vaccines has been reported^8,9^ but data from observational cohorts have yet to show an increased incidence.^10,11^ Hence it currently remains unclear if CVT is indeed associated with mRNA-based SARS-CoV-2 vaccines.

In Singapore, the national vaccination program against SARS-CoV-2 commenced on December 30, 2020, and used mRNA-based SARS-CoV-2 vaccines. The program began initially with BNT162b2 (Pfizer-BioNTech), and mRNA-1273 (Moderna) was subsequently included from March 12, 2021. The vaccination program began with essential workers and gradually extended to the population based on age, starting with residents aged 70 years and above^12^ and subsequently to the rest of the population.^13^ Adenovirus vector-based vaccines were not used in Singapore. More than 5 million doses of mRNA-based SARS-CoV-2 vaccines have been administered, and more than 35% of the total population has been fully vaccinated as of June 21, 2021.^14^ This allowed us to conduct surveillance of CVT in the post-mRNA-based SARS-CoV-2 population.

Concern of adverse effects of SARS-CoV-2 vaccination is the most common reason contributing to vaccine hesitancy.^15^ Reports of CVT with adenovirus vector-based vaccines may have potentially affected vaccine acceptance.^16^ Therefore, by directly comparing CVT rates after mRNA-based SARS-CoV-2 vaccination to after SARS-CoV-2 infection, the perception of CVT after vaccination may be more appropriately contextualized, and vaccination rates may be potentially improved. In addition, because mRNA-based SARS-CoV-2 vaccination has been shown to prevent SARS-CoV-2 infection,^17,18^ determining the relative difference of CVT may expand the public health message that vaccinations potentially have additional secondary benefits. In this study, we aim to ascertain the risk of CVT following either SARS-CoV-2 infection vs mRNA-based SARS-CoV-2 vaccines. We also aim to describe the temporal association and clinical characteristics of CVT after SARS-CoV-2 infections and mRNA-based SARS-CoV-2 vaccination.

## Methods

A prospective observational cohort study was performed involving all public acute hospitals in Singapore from January 23, 2020, to August 3, 2021. All patients were treated in their respective hospital where they presented. All clinical data were deidentified at source by the site investigator and shared via a centralized database with all study investigators. The study protocol was approved by the SingHealth Centralized Institutional Review Board, which granted a waiver of consent to the use of deidentified data. We followed the Strengthening the Reporting of Observational Studies in Epidemiology (STROBE) reporting guideline.^19^

This study included consecutive CVT cases requiring hospitalization only if the onset of symptoms of CVT occurred after receipt of mRNA-based SARS-CoV-2 vaccination. Due to the variation of time interval between first and second doses of vaccines, we defined the period of exposure as within 6 weeks after receiving the last dose. CVT was considered incident to SARS-CoV-2 infection if it was diagnosed afterward but within 6 weeks of confirmation of SARS-CoV-2 infection, or within the same hospital admission. The rationale for using a 6-week interval from vaccine exposure was based on a similar study evaluating the association between vaccine-related complications following influenza vaccine.^20^ A similar 6-week interval from SARS-CoV-2 infection was adopted for comparison. In view of adopting a 6-week interval, the last date of vaccination or diagnosis of infection included was June 22, 2021, and the last date of CVT diagnosis was up to August 3, 2021. Only the first hospitalization detail was included if repeated hospitalizations for the same patient occurred. Admissions to private hospitals in Singapore were excluded. As the first case of SARS-CoV-2 infection was confirmed in Singapore on January 23, 2020, the total observation period for SARS-CoV-2 infection-related CVT was 18 months. As the national SARS-CoV-2 vaccination program was only initiated in Singapore on December 30, 2020, the total observation period for vaccination-related CVT was 31 weeks. All patients had symptomatic CVT confirmed by either computed tomography venogram or magnetic resonance venogram of the brain.

SARS-CoV-2 infection was defined by positive SARS-CoV-2 virus through reverse transcription–polymerase chain reaction (RT-PCR) via nasopharyngeal swab, or positive SARS-CoV-2 IgG using the Abbott SARS-CoV-2 IgG (Abbott Diagnostics) or the Elecsys Anti-SARS-CoV-2 assay (Roche Diagnostics), which are immunoassays designed against the nucleocapsid antibody of SARS-CoV-2. The vaccinated population included all who received any dose of either mRNA (BNT162b2 [Pfizer-BioNTech] or mRNA-1273 [Moderna]) vaccines. Other forms of SARS-CoV-2 vaccine were excluded. The national vaccination data were obtained with permission from the Singapore National Immunisation Registry and the national data for SARS-CoV-2 infection were obtained from the National Centre for Infectious Diseases, Singapore. Population-based data obtained were aggregated data without individual patient level information.

### Statistical Analysis

The IR of CVT associated with SARS-CoV-2 infection was calculated by dividing the observed CVT hospitalization episodes after SARS-CoV-2 infection by the total number of SARS-CoV-2 infection cases in Singapore as of June 22, 2021. The incidence rate (IR) of CVT associated with mRNA-based SARS-CoV-2 vaccinations was calculated by dividing the observed CVT hospitalizations occurring within 6 weeks of mRNA-based SARS-CoV-2 vaccinations with the total vaccinated population as of June 22, 2021. Each individual had a 6-week period of exposure risk. To compare the IR of CVT with SARS-CoV-2 infection and with mRNA-based SARS-CoV-2 vaccine, crude incidence rate ratios (IRRs) and 95% CIs were calculated using person-year data. Exact 2-sided Poisson test via Central method was performed to calculate the IRRs and CIs. We also compared CVT IR between the 2 available mRNA-based SARS-CoV-2 vaccines. Statistical analysis was conducted with R software (R Project for Statistical Computing). Statistical significance was considered when *P* < .05.

## Results

Among 62 447 individuals diagnosed with SARS-CoV-2 infection included in the study, 58 989 (94%) were male individuals; the median (range) age was 34 (0-102) years. Among 3 006 662 individuals who received at least 1 dose of mRNA-based SARS-CoV-2 vaccines, 1 626 623 (54.1%) were male; the median [range] age was 50 (12-121) years (Table 1). A total of 2 068 110 individuals (68.8%) received both doses of vaccine and 938 552 (31.2%) received only 1 dose; 2 283 221 (75.9%) received BNT162b2 (Pfizer-BioNTech) and 723 441 (24.1%) received mRNA-1273 (Moderna). The cumulative duration of exposure was 7205 person-years for the population of SARS-CoV-2 infections, 238 628 person-years for those who completed both doses, 108 294 person-years for those who only received a single dose, and 346 923 person-years for the whole vaccinated cohort.

**Table 1.** Summary of Study Population and Main Results

Characteristic	Participants, No. (%)	
	SARS-CoV-2 infection (n = 62 447)	SARS-CoV-2 vaccine (n = 3 006 662)
Sex		
Male	58 989 (94.5)	1 626 623 (54.1)
Female	3458 (5.5)	1 380 039 (45.9)
Age, median (range), y	34 (0-102)	50 (12-121)
Subtype of vaccines received		
BNT162b2	NA	2 283 221 (75.9)
mRNA-1273	NA	723 441 (24.1)
Total duration of exposure, person-years	7205	346 923
No. of CVT hospitalizations	6	9
Age, median (range), y	34 (27-40)	60 (46-76)
Male sex	6 (100)	7 (77.8)
Female sex	0	2 (22.2)
Annualized crude incidence rate of CVT hospitalization, per 100 000 person-years (95% CI)	83.3 (30.6-181.2)	2.59 (1.19-4.92)
Crude incidence rate ratio of CVT hospitalization (95% CI)	32.1 (9.4-101.0)	[Reference]
*P* value	<.001	NA

Abbreviations: mRNA, messenger RNA; NA, not applicable.

There were 6 hospitalized patients with CVT after SARS-CoV-2 infection (Table 2). The IR of CVT after SARS-CoV-2 infections was 83.3 (95% CI, 30.6–181.2) per 100 000 person-years. Of the 6 hospitalized patients with CVT, all were male; the median (range) age was 33.5 (27-40) years; only 1 (17%) patient presented initially with respiratory symptoms and developed neurological symptoms (headache) 3 days later. The remaining 5 patients (83.3%) presented initially with neurological manifestations (headache [n = 3], seizures [n = 2]) and were subsequently confirmed with SARS-CoV-2 infection through either positive RT-PCR (n = 3) or serology (n = 2), suggesting recent infection with SARS-CoV-2. Of note, 3 out of 5 patients (60%) in this cohort had positive SARS-CoV-2 nasopharyngeal RT-PCR swabs despite absence of respiratory symptoms. Only 1 patient had abnormal procoagulant investigations (reduced protein S, positive homocysteine and presence of lupus anticoagulant) and he was the only mortality in this cohort. None of the SARS-CoV-2 infection patients with CVT had thrombocytopenia.

**Table 2.** Description of Patients With Cerebral Venous Thrombosis After SARS-CoV-2 Infection

Sex/age	Comorbidities	Respiratory symptoms	Positive COVID-19 tests	Neurological symptoms	Days from COVID-19 symptoms	Location of CVT	Platelet count, per 10^9^/L, (range 150-450)	D-dimer (range <0.5μg/mL)	Normal thrombotic tests	Abnormal thrombotic tests	Treatment	mRS on discharge
M/31-40 y	None	None	Serology only	Seizures	NA	SSS, left transverse sinus, left sigmoid sinus, left IJV	265	4.38	Protein C, Protein S, anti-thrombin III, ACL, LAC, B2GP1, Factor V Leiden, G20210A	None	Warfarin	1
M/31-40 y	None	None	PCR	Headache, vomiting	NA	Right posterior condylar vein, right sigmoid sinus, right proximal IJV	305	4.3	ACL	None	None (had fracture of temporal bone)	0
M/31-40 y	None	Cough, pleuritic chest pain, fever	PCR	Headache	3	Left transverse and sigmoid sinus	187	<0.19	ACL	None	Dabigatran	0
M/21-30 y	None	None	Serology only	Headache	NA	SSS, transverse sinus, torcula, sigmoid sinuses	214	Not done	ACL, LAC, B2GP1	None	LMWH	1
M/31-40 y	None	None	PCR	Seizure	NA	Left transverse and sigmoid sinuses, left IJV	255	4.56	Protein C, antithrombin III, ACL, B2GP1	Protein S 53% (low) homocysteine elevated, LAC present	Surgical decompression, heparin or warfarin	6
M/21-30 y	Smoker	None	Serology only	Headache	NA	SSS, right transverse sinus, right sigmoid sinus, right IJV, bilateral cortical veins	338	<0.5	Protein C, Protein S, anti-thrombin III, ACL, LAC, B2GP1	None	Heparin or LMWH or warfarin	1

Abbreviations: ACL, anticardiolipin antibodies; B2GP1, B2 glycoprotein 1; CVT, cerebral venous thrombosis; IJV, internal jugular vein; G20210A, guanine substitution with adenine at position 20210 at the prothrombin gene; LAC, lupus anticoagulant; LMWH, low molecular weight heparin; M, male; mRS, modified Rankin scale; NA, not applicable; PCR, polymerase chain reaction; SARS-CoV-2, Severe Acute Respiratory Syndrome Coronavirus 2; SSS, superior sagittal sinus.

SI conversion factors: To convert platelet counts to ×10^3^/μL, multiply by 1.0; to convert d-dimer to nmol/L, multiply by 5.476.

There were 9 cases of CVT occurring after mRNA-based SARS-CoV-2 vaccination (Table 3). The median (range) age was 60 (46–76) years; 7 (77.8%) were male individuals and 2 (22.2%) were female individuals. Six (66.7%) received BNT162b2 (Pfizer-BioNTech) vaccine and 3 (33.3%) received mRNA-1273 (Moderna) vaccine. Seven (77.8%) developed CVT after completion of both doses of the vaccination. Only 2 patients underwent testing for antiplatelet factor 4 (PF4) antibodies and 1 was positive. Three patients underwent heparin-induced platelet aggregation (HIPA) testing, and all were normal. No patients with CVT after mRNA-based SARS-CoV-2 vaccination had thrombocytopenia, none developed COVID-19 after mRNA-based SARS-CoV-2 vaccination, and none had repeated hospitalizations.

**Table 3.** Description of Cerebral Venous Thrombosis Following Messenger RNA SARS-CoV-2 Vaccination

Vaccine received	Sex/age, years	Co-morbidities	Presenting symptoms	Days from 1st dose of vaccine	Days from 2nd dose of vaccine	Location of CVT	Platelet count, per 10^9^/L, (range 150-450)	Normal thrombotic tests	Abnormal thrombotic tests	COVID-19 test	Treatment	mRS on discharge
BNT162b2	F/61-70 y	Hypertension	Headache	30	9	Right transverse, sigmoid, IJV	383	HIPA, Protein C, Protein S, anti-thrombin III, ACL, LAC, B2GP1	None	PCR negative	Surgical decompression, heparin or warfarin	5
BNT162b2	M/71-80 y	Hypertension, hyperlipidemia, diabetes	Headache	28	7	Right transverse and sigmoid sinuses	292	LAC present, ACL, B2GP1, Protein C protein S	None	PCR negative	Heparin or warfarin	3
BNT162b2	F/51-60 y	Hypertension, hyperlipidemia, diabetes, TIA, family history of unprovoked PE	Right sided weakness and sensory loss, Seizure	29	8	SSS, right transverse sinus, right sigmoid sinus, right IJV, bilateral cortical veins	250	Protein C, Protein S, ACL, LAC, B2GP1	Antithrombin III = 55% (normal range: 80%-120%)	Not performed	LMWH or warfarin	2
BNT162b2	M/51-60 y	Hyperlipidemia	Headache, left sided weakness and vomiting	22	1	Right transverse, sigmoid sinus, IJV	300	Anti-PF4 antibody, Protein C, Protein S, anti-thrombin III, ACL, LAC, B2GP1	None	PCR and serology negative	LMWH or warfarin	2
BNT162b2	M/61-70 y	Hypertension, hyperlipidemia, diabetes	Seizures and left upper limb weakness	33	11	SSS, right transverse sinus, bilateral sigmoid sinuses	333	Protein C, Protein S, anti-thrombin III, ACL, LAC	None	PCR negative	LMWH warfarin	0
BNT162b2	M/51-60 y	DVT, scar epilepsy, encephalitis, hyperlipidemia	Headache, right sided weakness and seizure	19	NA	SSS, cortical veins	212	HIPA, Protein C, Protein S, ACL, B2GP1	Anti-PF4 antibody positive, Anti-thrombin III = 73% (normal range: 80%-130%).	PCR negative	Heparin or LMWH or dabigatran	3
mRNA-1273	M/41-50 y	Smoker	Right sided weakness and seizure	39	11	SSS, left transverse and sigmoid sinuses	224	Protein S, ACL, LAC, B2GP1 normal	Anti-thrombin III = 79% (normal range: 93%-125%), Protein C = 66% (normal range: 83%-144%)	PCR and serology negative	SSS thrombectomy	2
									D-dimer = 1.57 (normal range: <0.5ug/mL)		Heparin or LMWH or warfarin	
mRNA-1273	M/61-70 y	Hypertension, hyperlipidemia, ischemic stroke	Right sided weakness	29	1	Right frontal superior cerebral vein	244	LAC present, Protein C, Protein S, anti-thrombin III, ACL, B2GP1	None	PCR negative	LMWH	4
mRNA-1273	M/51-60 y	None	Headache, vomiting, diplopia	23	NA	SSS, right transverse and sigmoid sinus	251	HIPA, Protein C, Protein S, anti-thrombin III, ACL, LAC	None	PCR negative	LMWH then warfarin	0

Abbreviations: ACL, anticardiolipin antibodies; B2GP1, B2 glycoprotein 1; CVT, cerebral venous thrombosis; DVT, deep vein thrombosis; HIPA, heparin-induced platelet activation; ICH, intracranial hemorrhage; IJV, internal jugular vein; LAC, lupus anticoagulant; LMWH, low molecular weight heparin; mRS, modified Rankin scale; PCR, polymerase chain reaction; PF4, platelet factor 4 reactive antibodies; PE, pulmonary embolism; SARS-CoV-2, Severe Acute Respiratory Syndrome Coronavirus 2; SSS, superior sagittal sinus; TIA, transient ischemic attack.

SI conversion factor: To convert platelet counts to ×10^3^/μL, multiply by 1.0.

The IR of CVT hospitalizations was 2.93 per 100 000 person-years (95% CI, 1.18-6.04 per 100 000 person-years) after completion of both doses of mRNA-based SARS-CoV-2 vaccine and 2.59 per 100 000 person-years (95% CI, 1.19-4.92 per 100 000 person-years) after at least 1 dose of mRNA-based SARS-CoV-2 vaccine (Table 3). The median (range) interval to symptom onset was 28 (19-39) days from first dose of vaccine, and 7 (1-11) days from second dose of vaccine. The main presenting symptoms included focal neurological deficits (n = 7), headache (n = 5), and seizure (n = 4). Four patients had abnormal procoagulant investigations (reduced antithrombin III [n = 2], reduced protein C [n = 1] and positive lupus anticoagulant [n = 2]), and 1 had positive PF4 antibody but with optical density less than 1. None of the patients had thrombocytopenia.

The IRR of CVT hospitalizations with SARS-CoV-2 infection was 28.4 (95% CI, 7.9-98.6; *P* < .001) compared with those who received both doses of mRNA-based SARS-CoV-2 vaccine, and 32.1 (95% CI, 9.4-101.0; *P* < .001) compared with those who received at least 1 dose. The IRR of CVT hospitalization was 2.28 per 100 000 person-years (95% CI, 0.84-4.96 per 100 000 person-years) after at least 1 dose of BNT162b2 (Pfizer-BioNTech), and 3.59 per 100 000 person-years (95% CI, 0.74-10.5 per 100 000 person-years) after at least 1 dose of mRNA-1273 (Moderna). The relative risk of CVT hospitalization after mRNA-1273 (Moderna) compared with BNT162b2 (Pfizer-BioNTech) was 1.57 (95% CI, 0.26-7.39; *P* = .75) (Table 4).

**Table 4.** Summary of Messenger RNA-Based SARS-CoV-2 Vaccination Status in Singapore as of June 22, 2021

Characteristic	Vaccine, No.		Total
	BNT162b2 (Pfizer-BioNTech)	mRNA-1273 (Moderna)	
Participants, No. (cumulative duration of exposure, person-years)^a^			
Who received at least 1 dose of vaccine	2 283 221 (263 449)	723 441 (83 474)	3 006 662 (346 923)
Who received both doses of vaccine	1 733 611 (200 032)	334 499 (38 596)	2 068 110 (238 628)
Who only received 1 dose of vaccine	549 610 (63 417)	388 942 (44 878)	938 552 (108 294)
Total doses of vaccine administered, No.	4 016 832	1 057 940	5 074 772
Receiving only 1 dose			
No. of hospitalized CVT cases	1	1	2
Annualized incidence rate of hospitalized CVT cases per 100 000 person-years (95% CI)	1.58 (0.04-8.79)	2.23 (0.06-12.4)	1.85 (0.22-6.67)
Receiving both doses			
No. of hospitalized CVT cases	5	2	7
Annualized incidence rate of hospitalized CVT cases, per 100 000 person-years (95% CI)	2.50 (0.81-5.83)	5.18 (0.63-18.7)	2.93 (1.18-6.04)
Receiving at least 1 dose			
No. of hospitalized CVT cases	6	3	9
Annualized incidence rate of hospitalized CVT cases, per 100 000 person-years (95% CI)	2.28 (0.84-4.96)	3.59 (0.74-10.5)	2.59 (1.19-4.92)

Abbreviation: CVT, cerebral venous thrombosis.

^a^ Exposure was calculated based on 6 weeks after last dose of vaccine.

## Discussion

Our study found that the crude IR of CVT hospitalization after SARS-CoV-2 infection was approximately 28 to 32 times the IR of CVT after mRNA-based SARS-CoV-2 vaccines. These results suggest that the associated risk of CVT with SARS-CoV-2 infection was much higher compared with mRNA-based SARS-CoV-2 vaccination. The crude IR of CVT in COVID-19 infection observed in the study (83 per 100 000 person-years) was much higher than the risk of CVT reported in the general population (1.3-2.0 per 100 000 person-years),^21^ again supporting the observation that CVT is strongly associated with SARS-CoV-2 infection. However, due to the observational nature of the study where randomization was not possible, the group who experienced SARS-CoV-2 infection may have other unmeasured differences compared with the healthy population that received vaccination. As confounding and biases cannot be ruled out, these results only suggest an association and do not imply causation.

The crude IR of CVT in our vaccinated population who received 2 doses of mRNA-based SARS-CoV-2 vaccine was 2.93 per 100 000 person-years (95% CI, 1.18-6.04 per 100 000 person-years). The CI falls within the baseline IR of CVT in Australia and the United States (1.3-2.0 per 100 000 person-years),^21,22^ suggesting the association of CVT with mRNA-based SARS-CoV-2 vaccine may be similar to background risk. However, due to the rarity of CVTs events, and the lack of direct comparison to baseline risk in our population, true association of CVT due to the vaccines could not be definitively concluded. We observed that the crude IR of CVT hospitalizations after mRNA-1273 (Moderna) was not statistically higher compared with BNT162b2 (Pfizer-BioNTech) as the rarity of the CVT events limit any meaningful comparisons between the 2 mRNA-based SARS-CoV-2 vaccines. Moreover, the reported IR of CVT in other populations appear to fall within the CIs of both BNT162b2 (Pfizer-BioNTech) and mRNA-1273 (Moderna), therefore supporting their safety profile.

CVT was observed in patients with SARS-CoV-2 infection without respiratory symptoms. Five out of 6 (83%) of our CVT cases with SARS-CoV-2 infection had no respiratory symptoms, suggesting the mechanism of CVT development may be independent of respiratory disease. The presence of positive serological tests, together with the negative nasopharyngeal SARS-CoV-2 RT-PCR tests in 3 (50%) of the patients, suggests a persistent prothrombotic risk of COVID-19 infection, similarly observed with SARS-CoV-2 related ischemic stroke.^23^ In contrast, the estimated IR of CVT in hospitalized patients with SARS-CoV-2 with respiratory symptoms was at 8.8 per 10 000 persons in 3 months, or a calculated 352 per 100 000 person-years,^4^ suggesting that the risk of CVT in symptomatic SARS-CoV-2 patients may be much higher. As CVT was the first manifestation of COVID-19 in these 5 patients, there was uncertainty of the actual onset of COVID-19 infection. Regardless, the association of CVT in asymptomatic COVID-19 infection may be higher, as compared with the general population.

CVT remains very rare compared with other venous thrombotic complications, such as pulmonary embolism (PE) and deep vein thrombosis (DVT), in COVID-19^24^ or mRNA SARS-CoV-2 vaccines.^10^ PE and DVT have been described in much higher frequency (approximately 20%) in hospitalized and severe COVID-19 infections.^25^ In contrast to after COVID-19 infections, no increase in PE has been observed after mRNA-based SARS-CoV-2 vaccines.^26^ More recently, the Delta variant of SARS-CoV-2 has been observed to require more hospitalizations than the Alpha variant,^27^ suggesting a theoretical possibility of a higher rate of CVT and a difference in latency from infection. However, the absence of information on the SARS-CoV-2 variants in our cohort limit any further conclusion and warrant further investigation.

All patients with SARS-CoV-2–related CVT in our cohort were young male individuals (aged 27 to 40 years). This was primarily because of the majority (approximately 77%) of the COVID-19 cases in Singapore were young male migrant workers living in dormitories.^28^ In contrast, mRNA-based SARS-CoV-2 vaccinations–related CVT occurred in predominantly older male individuals aged 46 to 76 years. The prioritization of vaccines for older individuals within the population^12,13^ likely resulted in a higher median age of CVT associated with mRNA-based SARS-CoV-2 vaccinations. However, the observed male predominance was in contrast with the female predisposition for CVT seen in other populations^21,22^ and despite an almost balanced sex distribution of mRNA-based SARS-CoV-2 vaccinations in Singapore (eTable in the Supplement).

The majority (83%) of the SARS-CoV-2–related CVT had normal prothrombotic evaluation, suggesting no alternative excessive risk of thrombosis, other than SARS-CoV-2 infection. In contrast, 3 out of 9 (33%) of the patients with vaccine-related CVT had abnormal prothrombotic evaluation, suggesting an underlying predisposition for thrombosis. However, as the majority did not have underlying prothrombotic conditions, and with similar IR of CVT compared with the general population, it remains uncertain if the CVT was related to the mRNA SARS-CoV-2 vaccines.

CVT occurring after adenoviral vector-based SARS-CoV-2 vaccines^6,7^ is often associated with thrombocytopenia and the pathophysiology of thrombosis has been postulated to be due to vaccine-induced immune thrombotic thrombocytopenia (VITT).^29,30^ VITT is a syndrome of thrombosis, in particular of the cerebral venous sinuses, with thrombocytopenia and presence of antibodies to PF4. These antibodies, similar to heparin-induced thrombocytopenia antibodies but without prior heparin exposure, develop with administration of modified adenovirus vector-based SARS-CoV-2 vaccines. However, the platelet counts of all CVTs in our series after mRNA SARS-CoV-2 vaccines were normal, conflicting with other observations of thrombosis with thrombocytopenia associated with mRNA SARS-CoV-2 vaccines,^31^ hence the association of CVT after mRNA SARS-CoV-2 vaccines remain uncertain. Although not universally tested, 1 patient had positive PF4 antibodies (with a low optical density of <1) with negative HIPA assay, indicating that these antibodies are unlikely to be pathogenic. This is corroborated by studies that showed that low titers of PF4 antibodies (with optical density between 0.5 and 1) can occur after vaccination with mRNA vaccines, but the majority of these antibodies likely have minor clinical importance.^32^

Two patients tested positive for lupus anticoagulant post-mRNA vaccination. Although the clinical importance of a transient lupus anticoagulant positivity is an epiphenomenon with no associated risk for thrombosis, it is unclear if this lupus anticoagulant detected postvaccination is similar to that of antiphospholipid antibodies developing during acute COVID-19. Such antibodies are found to be mainly directed against β_2_GPI but display an epitope specificity different from those antibodies in antiphospholipid syndrome which predispose to an increased thrombotic risk.^33^

There are other observed differences between CVT associated with mRNA-based SARS-CoV-2 vaccines compared with those associated with adenoviral vector-based vaccines. First, the onset of CVT after mRNA-based SARS-CoV-2 vaccine was approximately 28 days (range: 19-39 days) after the first dose, and 7 days after the second dose. In contrast, the onset of CVT after adenoviral vector-based vaccines was approximately 7 to 10 days after the first dose.^30^ Second, 80% of the patients in our series were male, which in contrast to VITT, predominantly affected female individuals.^30^ These epidemiological observations suggest differing mechanisms of development of CVT between the 2 types of SARS-CoV-2 vaccines.

### Limitations

The main limitation of our study was the absence of a suitable comparator group, such as a local historical population-based baseline CVT rates, or contemporaneous CVT cases unrelated to SARS-CoV-2 infection or mRNA SARS-CoV-2 vaccines. Furthermore, the case identification of CVT cases were purely hospital-based, which may not be representative of the whole population. A hospital-based cohort would have likely missed CVT with mild symptoms treated in the ambulatory setting, hence underestimating true CVT incidence. Our study did not include private hospitals in Singapore, which account for approximately 20% of all inpatient admissions, hence introducing potential selection bias and also underreporting of CVT cases. The high proportion of asymptomatic SARS-CoV-2 infection in our study suggests the possibility of underdiagnosis of SARS-CoV-2 infection-related CVT, therefore underestimating the true IR of CVT related to SARS-CoV-2 infection. Conversely, because of the potential underestimation of true SARS-CoV-2 infections in the population, results may represent an overestimation. As vaccines were prioritized to older residents and only 35% of the population received the vaccine, these represent potential sources of bias. The disparity in age between the younger SARS-CoV-2 infection and the older vaccinated population suggest inherent differences. Furthermore, the group with SARS-CoV-2 infection may have other unmeasured differences compared with the healthy population who received mRNA-based SARS-CoV-2 vaccine. The crude IR and IRR presented were unadjusted for differences between groups as only aggregated source data were available, hence must be interpreted with caution.

## Conclusions

Although rare, the incidence rate of SARS-CoV-2 infection-related CVT was significantly higher than mRNA-based SARS-CoV-2 vaccine-related CVT. CVT remains rare after mRNA-based SARS-CoV-2 vaccines, reinforcing its safety profile.

## Notes

Supplement. **eTable.** National Vaccination data in Singapore as of 22 June 2021 from National Immunisation Registry, Singapore.
