# Supplementary material for: Incidence of Cerebral Venous Thrombosis Following SARS-CoV-2 Infection vs mRNA SARS-CoV-2 Vaccination in Singapore
Source: JAMA Netw Open. 2022 Mar 17;5(3):e222940. doi: 10.1001/jamanetworkopen.2022.2940 (PMC8931554; doi:10.1001/jamanetworkopen.2022.2940)
Supplement: Supplement. — eTable. National Vaccination data in Singapore as of 22 June 2021 from National Immunisation Registry, Singapore. [file jamanetwopen-e222940-s001.pdf]

Supplemental Online Content

Tu TM, Yi SJ, Koh JS, et al. Incidence of cerebral venous thrombosis following SARS-CoV-2 infection vs mRNA SARS-CoV-2 vaccination in Singapore. *JAMA Netw Open.* 2022;5(3):e222940. doi:10.1001/jamanetworkopen.2022.2940

eTable. National Vaccination Data in Singapore as of 22 June 2021 From National Immunisation Registry, Singapore

This supplemental material has been provided by the authors to give readers additional information about their work.

© 2022 Tu TM et al. *JAMA Network Open.*

eTable. National Vaccination data in Singapore as of 22 June 2021 from National Immunisation Registry, Singapore*

	First dose administered	Second dose administered	Total number of doses administered
Sex			
Females, number (%)	1,387,119 (46.0)	975,570 (47.2)	2,362,689 (46.5)
Males, number (%)	1,626,623 (54.0)	1,092,541 (52.8)	2,719,164 (53.5)
Age group			
12-19, number (%)	244,318 (8.1)	19,821 (0.96)	264,139 (5.2)
20-29, number (%)	341,146 (11.3)	196,149 (9.5)	537,295 (10.6)
30-39, number (%)	392,065 (13.0)	214,727 (10.4)	606,792 (11.9)
40-49, number (%)	691,297 (22.9)	402,341 (19.5)	1,093,638 (21.5)
50-59, number (%)	557,015 (18.5)	497,451 (24.1)	1,054,466 (20.7)
60-69, number (%)	469,509 (15.6)	442,401 (21.4)	911,910 (17.9)
70-79, number (%)	230,950 (7.7)	216,131 (10.5)	447,081 (8.8)
80 and above, number (%)	87,442 (2.9)	79,090 (3.8)	166,532 (3.3)
Total doses administered	3,013,742	2,068,111	5,081,853

*Vaccination data includes 7081 doses of Sinovac-CoronaVac COVID-19 vaccine (Sinovac) (7080 first doses and one second dose) made publicly available in Singapore since 18 June 2021.

© 2022 Tu TM et al. *JAMA Network Open.*
